# Transactions of the College of Physicians of Philadelphia

**Published:** 1875-10

**Authors:** 


TRANSACTIONS OF THE COLLEGE OF PHYSICIANS OF PHILADELPHIA. Volume the Eighth. Book : pp. 192.

Among other valuable articles is a full report of an Autopsy on the bodies of Chang and Eng Bunker, commonly known as the Siamese Twins.

